# Associations between fatigue, physical fitness, and physical activity in patients with inflammatory bowel disease

**DOI:** 10.1093/crocol/otag052

**Published:** 2026-06-12

**Authors:** Karlijn Demers, Bart C Bongers, Sander M J van Kuijk, Guy Plasqui, Zlatan Mujagic, Daisy M A E Jonkers, Marieke J Pierik, Laurents P S Stassen

**Affiliations:** Department of Surgery, Maastricht University Medical Center+, Maastricht 6229 HX, the Netherlands; Department of Gastroenterology-Hepatology, Maastricht University Medical Center+, Maastricht 6229 HX, the Netherlands; Department of Surgery, Institute of Nutrition and Translational Research in Metabolism (NUTRIM), Maastricht University, Maastricht 6200 MD, the Netherlands; Department of Gastroenterology-Hepatology, Institute of Nutrition and Translational Research in Metabolism (NUTRIM), Maastricht University, Maastricht 6200 MD, the Netherlands; Department of Surgery, Institute of Nutrition and Translational Research in Metabolism (NUTRIM), Maastricht University, Maastricht 6200 MD, the Netherlands; Department of Nutrition and Movement Sciences, Institute of Nutrition and Translational Research in Metabolism (NUTRIM), Maastricht University, Maastricht 6200 MD, the Netherlands; Department of Clinical Epidemiology and Medical Technology Assessment (KEMTA), Maastricht University Medical Center +, Maastricht 6202 AZ, the Netherlands; Department of Nutrition and Movement Sciences, Institute of Nutrition and Translational Research in Metabolism (NUTRIM), Maastricht University, Maastricht 6200 MD, the Netherlands; Department of Gastroenterology-Hepatology, Maastricht University Medical Center+, Maastricht 6229 HX, the Netherlands; Department of Gastroenterology-Hepatology, Institute of Nutrition and Translational Research in Metabolism (NUTRIM), Maastricht University, Maastricht 6200 MD, the Netherlands; Department of Gastroenterology-Hepatology, Institute of Nutrition and Translational Research in Metabolism (NUTRIM), Maastricht University, Maastricht 6200 MD, the Netherlands; Department of Gastroenterology-Hepatology, Maastricht University Medical Center+, Maastricht 6229 HX, the Netherlands; Department of Gastroenterology-Hepatology, Institute of Nutrition and Translational Research in Metabolism (NUTRIM), Maastricht University, Maastricht 6200 MD, the Netherlands; Department of Surgery, Maastricht University Medical Center+, Maastricht 6229 HX, the Netherlands; Department of Surgery, Institute of Nutrition and Translational Research in Metabolism (NUTRIM), Maastricht University, Maastricht 6200 MD, the Netherlands

**Keywords:** fatigue, physical fitness, exercise, Crohn disease, ulcerative colitis

## Abstract

**Aim:**

Fatigue is a common and debilitating symptom in patients with inflammatory bowel disease (IBD) and may be linked to physical fitness and physical activity. However, their interrelationships are not fully understood. More objective knowledge is needed to guide physical interventions to reduce fatigue. This study aimed to investigate the association of physical fitness and physical activity with fatigue in patients with IBD. Additionally, the association between physical fitness and physical activity was investigated.

**Methods:**

Patients with IBD in remission or with mild-to-moderate disease activity were included. Fatigue was defined as a score of ≥35 on the subjective fatigue subscale of the checklist individual strength. Physical fitness measurements were performed using gold standard methodologies for body composition, cardiorespiratory fitness, muscular strength, muscular endurance, and flexibility. Physical activity was quantified using accelerometry.

**Results:**

In total, 53 patients were included, of whom 17 (32.1%) experienced fatigue. Fatigued patients exhibited a higher fat-mass index (*P *= .014), lower cardiorespiratory fitness (*P *= .017), and less quadriceps muscular endurance (*P *= .026, *P *= .008). No differences were observed in physical activity. Multiple linear regression showed that fat-mass index and cardiorespiratory fitness independently predicted fatigue (*P *= .029, *P *= .012). Significant correlations were found between fatigue and physical fitness, but not with physical activity.

**Conclusions:**

Reduced physical fitness was associated with fatigue in patients with IBD, suggesting that enhancing physical fitness could help alleviate fatigue. Physical activity was not associated with fatigue or physical fitness, indicating that simply increasing physical activity may have limited impact on reducing fatigue or improving physical fitness in this population.

## Introduction

Fatigue is one of the most common and debilitating symptoms in patients with inflammatory bowel disease (IBD) and often persists even in the absence of ongoing mucosal inflammation.^1^^,^^2^ It has a profound impact on quality of life and work productivity and ranks high among symptom concerns in patients.^3–6^ Nevertheless, it is difficult to treat in daily practice, in part because the underlying etiology of fatigue in patients with IBD remains poorly understood. It is assumed that fatigue arises from a complex interplay of factors, involving ongoing inflammation, anemia, nutritional deficiencies, altered metabolomics profiles, psychological comorbidity, sleep disturbances, microbiome alterations, but also lifestyle, smoking, diet, and physical activity.^7^

Previous studies indicate that higher levels of physical fitness and physical activity are associated with reduced fatigue in patients with IBD.^8–12^ Therefore, improving physical fitness and physical activity may have a potential role in managing fatigue among these patients. Components of physical fitness related to health are often referred to as health-related physical fitness, encompassing the key components body composition, cardiorespiratory fitness, muscular strength and endurance, and flexibility.^13^ Fatigue, but also other barriers such as abdominal pain, the fear of exacerbating symptoms, and psychological factors are thought to limit patients’ physical activity.^14–18^ Prolonged periods of physical inactivity can, in turn, result in overall deconditioning and reduced physical fitness, which could be worsened by an increased metabolic demand caused by chronic inflammation and immune system activation, malnutrition, and medication use in IBD.^19–23^ As physical fitness decreases, everyday activities become physically more demanding, potentially leading to increased feelings of fatigue and reduced energy levels.^24^^,^^25^ Additionally, patients may find themselves less motivated to engage in physical activities, further perpetuating the cycle of reduced fitness and increased fatigue.

Although this concept highlights potential areas for therapeutic intervention for managing fatigue, research on the relationship between fatigue, physical fitness, and physical activity in patients with IBD remains limited. More objective knowledge of these relationships is warranted to support the development of effective physical activity or physical exercise training interventions to reduce fatigue. Therefore, this study aimed to investigate the association of fatigue with health-related physical fitness components (ie, body composition, cardiorespiratory fitness, muscular strength, muscular endurance, and flexibility) and physical activity in patients with IBD using gold standard methodologies. Additionally, the relationship between health-related physical fitness components and physical activity was investigated.

## Materials and methods

### Study design and patient population

For this cross-sectional study, patients with Crohn’s disease (CD) and ulcerative colitis (UC) were consecutively recruited from the gastroenterology-hepatology outpatient clinic of the Maastricht University Medical Center+ between August 2022 and October 2023. Patients were eligible if they were aged 18 years or older, had an American Society of Anesthesiologists (ASA) Physical Status ≤ II, and were in remission or experienced mild-to-moderate clinical disease activity. At the time of inclusion, clinical disease activity was assessed using the Harvey–Bradshaw index (HBI) for CD and the simple clinical colitis activity index (SCCAI) for UC. Remission was defined by an HBI score below 5 or an SCCAI score below 3, mild disease activity by HBI scores ranging from 5 to 7 or SCCAI scores from 3 to 5, and moderate disease activity by HBI scores between 8 and 16 or SCCAI scores from 6 to 11.^26–29^ Exclusion criteria comprised: (1) severe clinical disease activity (ie, HBI > 16 or SCCAI > 11), (2) injuries or (neuro)muscular, rheumatic, or orthopedic conditions that could potentially affect study evaluations, (3) pregnant or breastfeeding women, given the marked physiological and body composition changes occurring during pregnancy and lactation, and (4) athletes competing at elite or competitive levels.^30^ Additionally, the physical activity readiness questionnaire was used to screen for any health risks that might result from performing exercise activities.^31^

### Data collection

Demographic and clinical data were collected from electronic patient records, including sex, age, comorbidities, disease entity, disease duration, Montreal classification, medication use, and previous intestinal resections. Fecal calprotectin and hemoglobin values were collected if available within an 8-week period of the study. Fecal calprotectin values under 250 μg/g were indicative of biochemical remission.^32^ The presence of anemia was defined as a hemoglobin level of <8.1 mmol/L for males and <7.5 mmol/L for females, according to the World Health Organization definition.^33^ Additionally, patients were queried about their smoking status, level of education, and current employment situation during the study visit.

### Fatigue assessment

Fatigue was assessed using the validated Dutch checklist individual strength (CIS).^34^ This questionnaire, composed of 20 items, evaluates 4 dimensions of fatigue experienced in the past 2 weeks: subjective fatigue, concentration, motivation, and activities. Fatigue was defined as a score of 35 or higher on the subjective fatigue subscale, based on a large cohort of patients with chronic fatigue syndrome in comparison with the general population.^35^

### Physical fitness assessment

Participants performed tests for body composition, cardiorespiratory fitness, muscular strength, muscular endurance, and flexibility. All tests were carried out by one trained clinical researcher (K.D.). Body mass was measured to the nearest 0.1 kg using an electronic scale. Participants were barefoot and wore light clothing. Body height was determined to the nearest 0.01 cm with a stadiometer. BMI was calculated by dividing body mass (in kg) by the square of body height (in meters).

Body composition was evaluated using the deuterium oxide (D_2_O) dilution technique.^36^ Patients collected a background urine sample before drinking around 75 mL of deuterium-enriched (∼4.3%) water. The next morning, after an overnight equilibration, a second urine sample of the second voiding was collected. The isotope enrichment in these samples was analyzed using isotope ratio mass spectrometry (IRMS, Thermo Scientific, Delta V). A hydration fraction of fat-free mass (FFM) of 0.73 was applied to calculate the FFM and fat mass (FM).^37^ Fat-free mass index (FFMI) and fat mass index (FMI) were then obtained by dividing FFM and FM by height squared, and are expressed in kg/m^2^.^38^

Cardiorespiratory fitness was measured with a cardiopulmonary exercise test (CPET) on a cycle ergometer, assessing oxygen uptake at peak exercise (VO_2peak_). Patients wore an oro-nasal facemask (V2 series, Hans Rudolph Inc.) for respiratory gas analysis, which was connected to an ergospirometry system (Vyntus CPX, Vyaire Medical Inc.) calibrated for respiratory gas analysis and volume measurements. In addition, patients were fitted with a 12-lead electrocardiogram (Vyntus ECG, Vyaire Medical Inc.) for heart rate and rhythm monitoring during the test. Blood pressure was measured at 2-minute intervals (Tango M2, SunTech Medical Inc.), and SpO_2_ was continuously monitored at the ear (8000Q2 Oximetry Sensor, NONIN Medical Inc.). The test started with a 2-minute seated rest, followed by a 3-minute warm-up of unloaded cycling. Subsequently, the work rate increased continuously in a ramp-like manner, tailored to the estimated fitness level of each participant, aiming for maximal exertion in 8-12 minutes. Patients had to maintain a pedaling frequency of 70-80 revolutions per minute, with the test terminating when the pedaling frequency dropped below 60 revolutions per minute, despite verbal encouragement. VO_2peak_ was calculated as the average value over the final 30 seconds before the end of the test and was considered (near-)maximal if the respiratory exchange ratio at peak exercise (RER_peak_) exceeded 1.10 and/or the maximal heart rate reached over 85% of the predicted maximum (ie, 208—0.7 × participant’s age). VO_2peak_ was expressed in ml/kg/min and analyzed as a continuous variable, with values typically ranging from approximately 15-55 mL/kg/min depending on age, sex, and fitness level.^39^

Muscular strength and endurance of the quadriceps and hamstring muscles of the dominant leg were assessed using the Biodex System 4 Pro dynamometer (Biodex Medical System Inc). Patients were seated in the dynamometer chair, secured with straps to minimize compensatory movements, and their dominant leg was attached to the Biodex system just above the ankle joint. The testing protocol included 3 sets of maximal repetitions at varying speeds: 3 at 60°/s, 5 at 90°/s, and 30 at 180°/s, with 60-second rests between sets. A single repetition consisted of a maximal isokinetic contraction of the quadriceps followed by the hamstrings. Maximal isokinetic peak torque, expressed in Newton-meters (Nm) and corrected for body mass, was obtained by averaging the 2 highest torques achieved per speed and was analyzed as a continuous variable, where higher values reflect greater maximal muscle strength. Muscular endurance was assessed with 2 fatigue indices derived from the 30 repetitions at 180°/s.^40^^,^^41^ The peak torque fatigue index, measured in Nm, assesses the ability to sustain maximal muscular strength and was calculated with the formula: (100 − (sum of peak torque from last 5 repetitions/sum of peak torque from the highest consecutive 5 repetitions) *×* 100). In addition, the work fatigue index, measured in Joule, measures the decline in total energy output and was calculated with the formula: (100 − (work performed during last 10 repetitions/work performed during first 10 repetitions) *×* 100). Both indices were expressed as continuous values, with 0 reflecting no decline and higher values indicating greater fatigue and thus reduced muscular endurance.

Flexibility was assessed using the sit-and-reach test, which measures the flexibility of the lower back and hamstrings. Participants sat on a mat with extended legs and bare feet positioned against a measuring box, reaching forward as far as possible while keeping their arms and knees straight. To minimize the injury risk, rapid or jerky movements were discouraged. The test was performed 3 times, with the greatest distance counting rounded down to the nearest whole centimeter. If a participant was unable to reach the box, a value of zero centimeters was noted.

### Physical activity assessment

Physical activity was measured with the compact (35 × 35 × 10 mm, 11 g) and waterproof MOX Activity Monitor (MOX; Maastricht Instruments).^42^ The MOX accelerometer records acceleration data in 3 axes at a 25 Hz sampling rate. The integrated algorithm of the MOX quantifies the extent of physical activity by calculating the total counts per second (cps) over 2-second intervals.^42^ These cps reflect the intensity of physical activity, with higher values indicating more intense or frequent movements. A threshold of 7 cps was used to differentiate dynamic physical activity from a sedentary or standing position. A sensor orientation threshold of 0.8 g discriminated between a standing and sedentary position, utilizing the gravitational vector relative to the sensor’s orientation to determine the posture. The MOX was attached by the researcher (K.D.) to the anterior upper thigh of the participant, about 10 cm above the knee, using a custom-made, double-sided, hypoallergenic, and waterproof patch. Patients were instructed to wear the accelerometer continuously for 7 consecutive days and to maintain their usual daily physical activity routines. Given the circadian rhythm’s impact on physical activity levels throughout the day, only days with 24 hours of physical activity measurement, with a minimum wear-time of 5 days, were considered for the analysis of daily averages of physical activity.

To calculate the average counts per day, the number of cps for every 2 seconds was summed for each 24-hour period and then averaged over the total number of days the MOX device was worn by the participants. For the activity categories, the total minutes spent daily in each specific category (ie, sedentary, standing, and dynamic) were calculated. The number of sit-to-stand transitions per day was calculated as the number of changes in posture from a sedentary state to either standing or dynamic activity. Additionally, daily patterns of physical activity were examined by calculating counts per hour, achieved by summing the cps for every 2 seconds for each hourly interval throughout the day, followed by averaging these counts based on the frequency each specific hour was recorded during the week.

### Statistical analysis

Statistical analysis was performed using IBM SPSS Statistics (IBM Corp.). Descriptive statistics were used to present baseline characteristics, measures of physical fitness tests, and physical activity monitoring. Because the numerical variables were non-normally distributed, they are presented as median (1st quartile [Q1], 3rd quartile [Q3]), and categorical variables as number and percentage of patients. Missing data for specific physical fitness or physical activity measures were excluded only from the corresponding analyses. Measures for body composition (FMI, FFMI), cardiorespiratory fitness (VO_2peak_), and flexibility (reached distance with the sit-and-reach test) were also expressed as percentages of predicted values based on age- and sex-specific normative values derived from healthy reference populations.^31^^,^^38^^,^^39^^,^^43^ Percentages of predicted values were calculated using the formula: (measured value/predicted value) × 100%. Normative values for FMI and FFMI were obtained from Schutz et al., while those for VO_2peak_ were sourced from the Lowlands Fitness Registry by van der Steeg et al. Normative values for flexibility were derived from the Canadian Society for Exercise Physiology. Percentages of predicted values were not calculated for muscular strength and endurance since accurate normative values were not available.

Differences between patients with and without fatigue in demographic and clinical characteristics, measures of health-related physical fitness, and physical activity measures were analyzed using the Mann–Whitney *U* test for non-parametric numerical data, and Pearson’s chi-squared or Fisher’s exact test for categorical variables. Multiple linear regression analysis was used to examine whether measures of health-related physical fitness and physical activity predicted fatigue scores, while adjusting for predefined clinical confounders based on clinical expertise (ie, age, sex, active smoking, and fecal calprotectin). The primary focus of this analysis was to predict the CIS subjective fatigue subscale score. This ensured consistency with the measure used to classify participants as fatigued or non-fatigued. Additionally, analyses were conducted for the total CIS score and the other subscales (ie, concentration, motivation, and activity), with these results presented in the supplemental files. To examine the correlation between continuous variables for fatigue, physical fitness, and physical activity, Pearson’s *r* or Spearman’s *rho* were used, according to normality and the presence of outliers. Corrections for multiple testing were not applied due to the exploratory design of this study. This approach was chosen to minimize the likelihood of overlooking subtle but potentially meaningful associations that may warrant further investigation. Two-tailed *P-*value < .05 were considered statistically significant.

### Ethical considerations

This study was conducted in compliance with the Declaration of Helsinki and was approved by the Medical Ethical Committee of the Maastricht University Medical Center+ (registration no. 22-012) and registered in ClinicalTrials.gov (*NCT05482932*).^44^ All patients gave written informed consent prior to participation.

## Results

### Participants

In total, 53 patients with IBD (29 CD, 24 UC) were included, of whom 17 (32.1%) were classified as fatigued. Demographic and clinical characteristics of all participants are shown in Table 1. On a group level, fatigued patients had a significantly higher median BMI (27.9 [24.6, 32.1] kg/m^2^ vs. 25.0 [23.0, 27.0] kg/m^2^; *P *= .020), less frequently higher educated (higher vocational education or university [17.6% vs. 52.8%; *P *= .025], a higher prevalence of smoking [35.3% vs. 2.8%, *P *= .006], and more frequent clinical disease activity [52.9% vs. 11.1%, *P *= .002]). Body composition data was missing in 1 patient (1.9%) due to noncompliance with urine sampling, CPET data was missing in 1 patient (1.9%) because maximal effort was not achieved, and physical activity data were missing in 3 patients (5.7%) owing to a knee injury, loss of the MOX device, or insufficient valid monitoring days.

**Table 1:** Demographic and clinical characteristics of all participants.

	Total	Fatigued	Non-fatigued	*P*-value
	(*n *= 53, 100%)	(*n *= 17, 32.1%)	(*n *= 36, 67.9%)	
**Age at inclusion, median (Q1, Q3)**	42.7 (32.2, 54.3)	43.1 (30.7, 52.1)	42.6 (31.8, 56.7)	.529
**Sex, female, *n* (%)**	24 (45.3)	9 (52.9)	15 (41.7)	.441
**Disease entity, *n* (%)**				.145
** CD**	29 (54.7)	12 (70.6)	17 (47.2)	
** UC**	24 (45.3)	5 (29.4)	19 (52.8)	
**CCI, *n* (%)**				1.000
** 0**	31 (58.5)	10 (58.8)	21 (58.3)	
** 1-2**	19 (35.8)	7 (42.2)	12 (33.3)	
** >2**	3 (5.7)	0 (0)	3 (8.3)	
**BMI (kg/m^2^), median (Q1, Q3)**	25.2 (23.2, 28,1)	27.9 (24.6, 32,1)	25.0 (23.0, 27.0)	**.020**
**BMI, *n* (%)**				.070
** Underweight (<18.5 kg/m^2^)**	0 (0)	0 (0)	0 (0)	
** Normal weight (18.5-24.9 kg/m^2^)**	22 (41.5)	4 (23.5)	18 (50.0)	
** Overweight (≥25.0 kg/m^2^)**	21 (39.6)	7 (41.2)	14 (38.9)	
** Obesity (≥30.0 kg/m^2^)**	10 (18.9)	6 (35.3)	4 (11.1)	
**Education level, *n* (%)**				**.025**
** Primary education**	0 (0)	0 (0)	0 (0)	
** Secondary education**	12 (22.6)	6 (35.3)	6 (16.7)	
** Intermediate vocational education**	19 (35.8)	8 (47.1)	11 (30.6)	
** Higher vocational education**	13 (24.5)	1 (5.9)	12 (33.3)	
** University**	9 (17.0)	2 (11.8)	7 (19.4)	
**Employment status, *n* (%)**				.085
** Working full-time**	19 (35.8)	3 (17.6)	16 (44.4)	
** Working part-time**	15 (28.3)	6 (35.3)	9 (25.0)	
** Studying**	8 (15.1)	3 (17.7)	5 (13.9)	
** Retired**	5 (9.4)	1 (5.9)	4 (11.1)	
** Sick leave**	3 (5.7)	2 (11.8)	1 (2.8)	
** Partially or fully unfit to work**	3 (5.7)	2 (11.8)	1 (2.8)	
**Current smoking status, *n* (%)**				**.006**
** Smokes daily**	4 (7.5)	4 (23.5)	0 (0)	
** Smokes occasionally**	3 (5.7)	2 (11.8)	1 (2.8)	
** Ex-smoker**	21 (39.6)	6 (35.3)	15 (41.7)	
** Never smoked**	25 (47.2)	5 (29.4)	20 (55.6)	
**Disease duration (years), median (Q1, Q3)**	11.3 (7.0, 22.7)	10.8 (8.8, 27.5)	11.3 (6.8, 20.6)	.620
**Montreal age at diagnosis, *n* (%)**				.360
** A1: ≤16 years**	7 (13.2)	4 (23.5)	3 (8.3)	
** A2: 17-40 years**	36 (67.9)	10 (58.8)	26 (72.2)	
** A3: >40 years**	10 (18.9)	3 (17.6)	7 (19.4)	
**Montreal disease location [CD], *n* (%)**				.588
** L1: ileal**	10 (34.5)	5 (41.7)	5 (29.4)	
** L2: colonic**	4 (13.8)	2 (16.7)	2 (11.8)	
** L3: ileocolonic**	15 (51.7)	5 (41.7)	10 (58.8)	
** Upper gastrointestinal disease**	3 (10.3)	1 (8.3)	2 (5.6)	1.000
**Montreal disease behavior [CD], *n* (%)**				.696
** B1: non-structuring, non-penetrating**	14 (48.3)	7 (58.3)	7 (41.2)	
** B2: structuring**	6 (20.7)	2 (16.7)	4 (23.5)	
** B3: penetrating**	9 (31.0)	3 (25.0)	6 (35.3)	
** Perianal disease**	7 (13.2)	3 (25.0)	4 (11.1)	1.000
**Montreal disease extension [UC], *n* (%)**				.480
** E1: proctitis**	1 (4.2)	0 (0)	1 (5.3)	
** E2: left-sided colitis**	9 (37.5)	3 (60.0)	6 (31.6)	
** E3: pancolitis**	14 (58.3)	2 (40.0)	12 (63.2)	
**Clinical disease activity, *n* (%)^a^**				**.002**
** Remission**	40 (75.5)	8 (47.1)	32 (88.9)	
** Mild disease activity**	12 (22.6)	8 (47.1)	4 (11.1)	
** Moderate disease activity**	1 (1.9)	1 (5.9)	0 (0)	
**Fecal calprotectin (μg/g), median (Q1, Q3)^b^**	38.0 (16.0, 158.3)	41.0 (20.0, 248.0)	33.0 (14.9, 117.0)	.390
**Biochemical disease activity, *n* (%)^b^**				1.000
** Remission**		13 (86.7)	28 (84.8)	
** Active disease**		2 (13.3)	5 (15.2)	
**Hemoglobin (mmol/L), median (Q1, Q3)^c^**	8.7 (8.2, 9.3)	8.7 (8.0, 9.3)	8.7 (8.4, 9.3)	.446
**Anemia, *n* (%)^d^**	1 (1.9)	1 (8.3)	0 (0)	.279
**Current IBD medication*, n* (%)**				.831
** None**	10 (18.9)	4 (23.5)	6 (16.7)	
** Mesalazine only**	5 (9.4)	2 (11.8)	3 (8.3)	
** (Topical) corticosteroids**	2 (3.8)	0 (0)	2 (5.6)	
** Immunomodulators**	5 (9.4)	2 (11.8)	3 (8.3)	
** Biologic agents**	31 (58.5)	9 (52.9)	22 (61.1)	
**Prior intestinal resection, *n* (%)**	12 (22.6)	4 (23.5)	8 (22.2)	1.000

Abbreviations: CCI, Charlson comorbidity index; CD, Crohn’s disease; HBI, Harvey–Bradshaw index; IBD, inflammatory bowel disease; Q1, 1st quartile; Q3, 3rd quartile; SCCAI, simple clinical colitis activity index; SD, standard deviation; UC, ulcerative colitis. Statistically significant values are highlighted in bold.

a Clinical disease activity according to the HBI for CD and the SCCAI for UC: remission was defined as HBI < 5 or SCCAI < 3, mild disease activity as HBI 5-7 or SCCAI 3-5, and moderate disease activity as HBI 8-16 or SCCAI 6-11.

b Available in *n* = 48; remission was defined as fecal calprotectin < 250 μg/g.

c Available in *n* = 43.

d Available in *n* = 43; defined as a hemoglobin level of <8.1 mmol/L for males and <7.5 mmol/L for females.

### Physical fitness and physical activity measures for fatigued and non-fatigued patients

Table 2 presents physical fitness and physical activity measures for all patients and for the fatigued and non-fatigued subgroups. Patients who reported fatigue demonstrated a higher total median FMI (9.8 [7.8, 12.0] kg/m^2^ vs. 7.1 [5.4, 9.7] kg/m^2^, *P *= .014) and a lower median VO_2peak_ (26.2 [21.6, 35.4] mL/kg/min vs. 40.3 [35.5, 52.8] mL/kg/min, *P *= .017) compared to those without fatigue. These between-group differences were also statistically significant when the absolute values were expressed as percentages of the predicted values. Fatigued patients also showed a significantly greater median quadriceps peak torque fatigue index (39.7 [29.9, 47.9] vs. 32.5 [25.9, 36.6], *P *= .026) and work fatigue index (46.6 [34.6, 50.8] vs. 35.8 [28.4, 42.4], *P *= .008), indicating less muscular endurance compared to non-fatigued patients. No significant differences were found in the muscular strength of the quadriceps and hamstring muscles, hamstring fatigue indices, and the sit-and-reach test between fatigued and non-fatigued patients.

**Table 2:** Physical fitness and physical activity measures of all patients, fatigued and non-fatigued patients.

Patient and clinical characteristics	Total	Fatigued	Non-fatigued	
	(*n *= 53, 100%)	(*n *= 17, 32.1%)	(*n *= 36, 67.9%)	*P*-value
**Physical fitness**				
** FMI (kg/m^2^), median (Q1, Q3)^a^**	8.0 (5.6, 10.0)	9.8 (7.8, 12.0)	7.1 (5.4, 9.7)	**.014**
** Percentage of predicted FMI, median (Q1, Q3)^a^**	138.5 (106.0, 178.0)	176.5 (138.5, 215.0)	122.5 (100.7, 169.6)	**.002**
** FFMI (kg/m^2^), median (Q1, Q3)^a^**	17.4 (16.0, 19.9)	17.4 (15.8, 20.8)	17.3 (16.0, 18.7)	.501
** Percentage of predicted FFMI, median (Q1, Q3)^a^**	98.5 (94.2, 108.6)	105.4 (93.6, 120.4)	97.3 (94.2, 103.6)	.136
** VO_2peak_ (ml/kg/min), median (Q1, Q3)^a^**	32.0 (25.2, 38.5)	26.2 (21.6, 35.4)	40.3 (35.5, 52.8)	**.017**
** Percentage of predicted VO_2peak_, median (Q1, Q3)^a^**	84.5 (69.9, 93.8)	71.1 (62.1, 87.9)	89.6 (79.7, 99.1)	**.002**
** Quadriceps peak torque, 60°/s (Nm/kg), median (Q1, Q3)**	1.8 (1.4, 2.3)	1.7 (1.2, 2.0)	1.8 (1.4, 2.4)	.189
** Quadriceps peak torque, 90°/s (Nm/kg), median (Q1, Q3)**	1.6 (1.2, 2.0)	1.5 (1.2, 1.8)	1.7 (1.2, 2.0)	.341
** Quadriceps peak torque, 180°/s (Nm/kg), median (Q1, Q3)**	1.2 (1.0, 1.5)	1.0 (1.0, 1.3)	1.2 (1.0, 1.6)	.295
** Hamstring peak torque, 60°/s (Nm/kg), median (Q1, Q3)**	1.0 (0.8, 1.2)	1.0 (0.7, 1.1)	1.0 (0.9, 1.3)	.170
** Hamstring peak torque, 90°/s (Nm/kg), median (Q1, Q3)**	1.0 (0.8, 1.2)	1.0 (0.6, 1.1)	1.0 (0.8, 1.2)	.370
** Hamstring peak torque, 180°/s (Nm/kg), median (Q1, Q3)**	0.8 (0.7, 1.0)	0.8 (0.6, 1.0)	0.8 (0.8, 1.0)	.123
** Quadriceps peak torque fatigue index, median (Q1, Q3)**	33.5 (28.8, 40.8)	39.7 (29.9, 47.9)	32.5 (25.9, 36.6)	**.026**
** Hamstring peak torque fatigue index, median (Q1, Q3)**	25.6 (18.0, 30.5)	25.8 (21.6, 34.9)	24.9 (17.8, 30.2)	.336
** Quadriceps work fatigue index, median (Q1, Q3)**	40.1 (30.8, 46.7)	46.6 (34.6, 50.8)	35.8 (28.4, 42.4)	**.008**
** Hamstring work fatigue index, median (Q1, Q3)**	33.8 (25.7, 39.9)	37.4 (31.2, 44.1)	32.1 (24.4, 38.0)	.076
** Sit-and-reach distance (cm), median (Q1, Q3)**	26.0 (18.5, 33.5)	27.0 (21.0, 35.0)	25.5 (16.3, 33.0)	.331
** Percentage of predicted sit-and reach distance, median (Q1, Q3)**	96.4 (70.3, 117.6)	100.0 (81.4, 119.0)	95.5 (66.7, 117.6)	.536
**Physical activity^b^**				
** Average total counts per day, median (Q1, Q3)**	158, 123.9 (135, 151.7, 179, 848.8)	144, 350.1 (129, 068.2, 170, 276.5)	162, 905.9 (138, 026, 194, 375.5)	.100
** Average daily minutes sedentary, median (Q1, Q3)**	1094.5 (1029.8, 1148.7)	1094.0 (1014.3, 1167.5)	1094.5 (1037.7, 1139.8)	.835
** Average daily minutes standing, median (Q1, Q3)**	180.8 (146.7, 218.5)	187.3 (136.3, 229.3)	176.7 (146.7, 218.5)	.868
** Average daily minutes dynamic activity, median (Q1, Q3)**	166.2 (139.1, 198.1)	155.9 (124.8, 198.6)	166.3 (145.4, 198.1)	.519
** Average daily sit-to-stand transitions, median (Q1, Q3)**	251.5 (222.6, 328.2)	270.3 (228.9, 340.6)	246.3 (216.6, 307.4)	.253

Abbreviations: CPET, cardiopulmonary exercise test; FMI, fat mass index; FFMI, fat-free mass index; Q1, 1st quartile; Q3, 3rd quartile; VO_2peak_, oxygen uptake at peak exercise. Statistically significant values are highlighted in bold.

a Available in *n *= 52.

b Available in *n *= 50.

**Figure 1:**
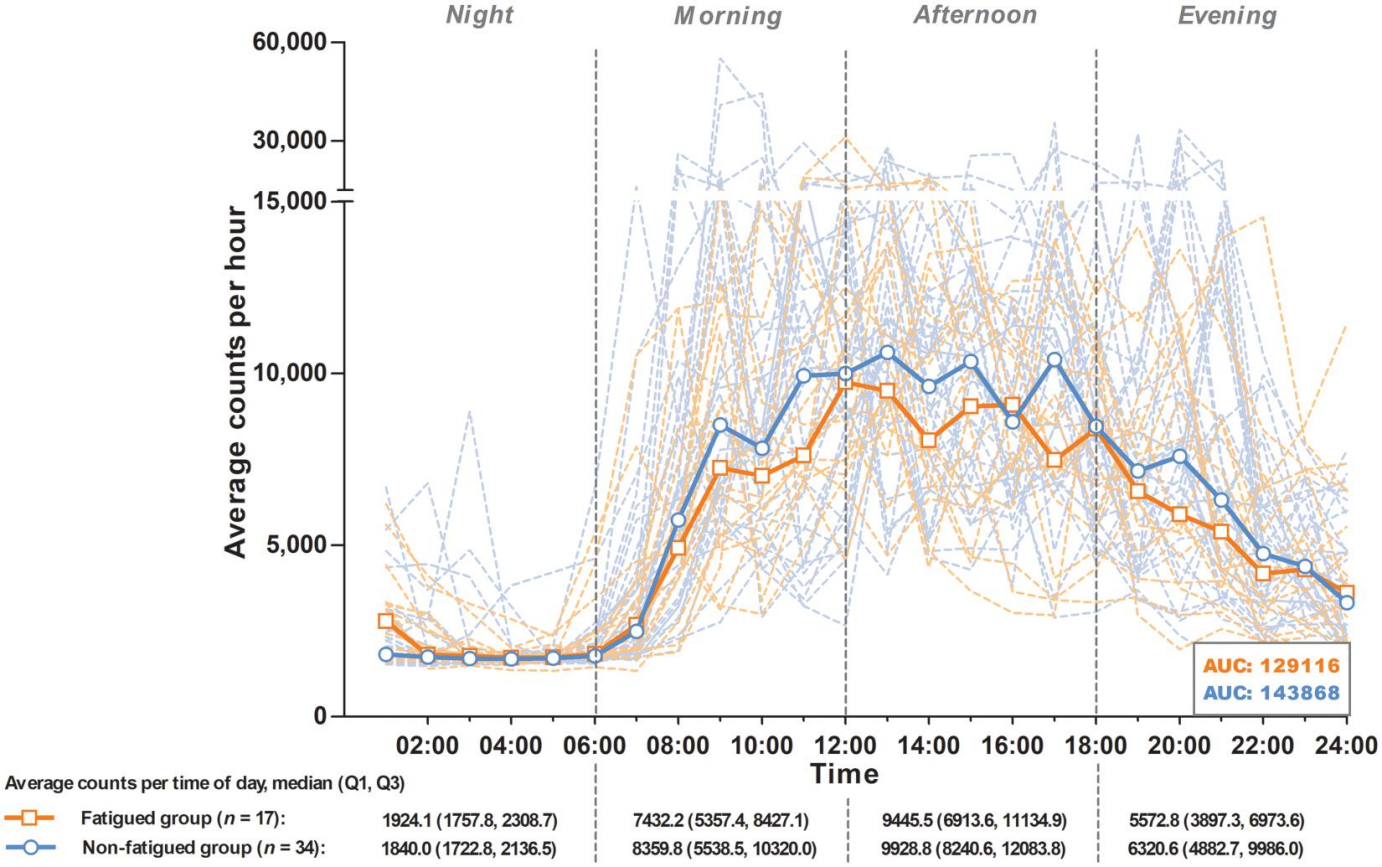
Physical activity patterns for fatigued patients and non-fatigued patients. AUC, area under the curve; cps, counts per second.

There were no statistically significant differences in physical activity parameters between fatigued and non-fatigued patients (Table 2). Daily physical activity patterns of fatigued and non-fatigued patients are visualized in Figure 1.

### Relationships between fatigue, physical fitness, and physical activity

Multiple linear regression analyses revealed that FMI (B 1.46 [95% confidence interval, CI 0.52-2.39], *P *= .029) and VO_2peak_ (B −0.48 [95% CI −0.82 to −0.14], *P *= .012) independently predicted the CIS subjective fatigue subscale score (Table 3). Additionally, the average daily minutes standing was associated with the CIS activity subscale score (B −0.02 [95% CI −0.04 to 0.00], *P *= .037; Table SII). However, none of the physical fitness components or measures of physical activity independently predicted the total CIS score, CIS concentration subscale score, or CIS activity subscale score (Tables SI, SIII, and SIV).

**Table 3:** Unadjusted and adjusted linear regression models predicting the CIS subjective fatigue subscale score.

	Unadjusted			Adjusted^a^		
	B	95% CI	*P*-value	B	95% CI	*P*-value
**Physical fitness**						
** FMI (kg/m^2^)^b^**	1.46	0.52 to 2.39	.**003**	1.18	0.13 to 2.22	**.029**
** FFMI (kg/m^2^)^b^**	0.57	−0.92 to 2.05	.448	0.81	−0.76 to 2.38	.305
** VO_2peak_ (mL/kg/min)^b^**	−0.48	−0.82 to −0.14	**.007**	−0.48	−0.84 to −0.11	**.012**
** Quadriceps peak torque, 60°/s (Nm/kg)**	−5.71	−11.64 to 0.21	.058	−4.73	−13.07 to 3.61	.259
** Quadriceps peak torque, 90°/s (Nm/kg)**	−5.19	−12.39 to 2.01	.154	−1.64	−12.989.69	.771
** Quadriceps peak torque, 180°/s (Nm/kg)**	−9.37	−19.03 to 0.28	.057	−8.91	−23.56 to 5.74	.227
**Hamstring peak torque, 60°/s (Nm/kg)**	−7.89	−18.39 to 2.62	.138	−11.85	−24.92 to 1.21	.074
**Hamstring peak torque, 90°/s (Nm/kg)**	−4.83	−16.506.84	.410	−7.26	−22.828.30	.352
** Hamstring peak torque, 180°/s (Nm/kg)**	−9.52	−24.51 to 5.47	.208	−14.51	−33.57 to 4.54	.132
** Quadriceps peak torque fatigue index**	0.38	0.05 to 0.711	**.026**	0.19	−0.11 to 0.49	.210
** Hamstring peak torque fatigue index**	0.28	−0.07 to 0.63	.118	0.13	−0.17 to 0.43	.390
** Quadriceps work fatigue index**	0.24	0.06 to 0.41	**.011**	0.15	−0.01 to 0.31	.057
** Hamstring work fatigue index**	0.33	0.06 to 0.60	**.019**	0.21	−0.03 to 0.44	.080
** Sit-and-reach test (cm)**	0.09	−0.28 to 0.47	.616	0.00	−0.32 to 0.85	.988
**Physical activity^c^**						
** Average daily counts per second**	0.00	00.00 to 0.00	.293	0.00	00.00 to 0.00	.069
** Average daily minutes sedentary**	0.00	−0.04 to 0.05	.972	0.00	−0.03 to 0.04	.823
** Average daily minutes standing**	0.00	−0.07 to 0.07	.965	0.00	−0.05 to 0.06	.905
** Average daily minutes dynamic activity**	−0.01	−0.09 to 0.08	.897	−0.02	−0.09 to 0.05	.550
** Average daily sit-to-stand transitions**	0.01	−0.02 to 0.04	.433	0.01	−0.02 to 0.04	.373

Abbreviations: CI, confidence interval; CPET, cardiopulmonary exercise test; FFMI, fat-free mass index; FMI, fat mass index; VO_2peak_, oxygen uptake at peak exercise. Statistically significant values are highlighted in bold.

a Adjusted for age, sex, active smoking, and fecal calprotectin.

b Available in *n *= 52.

c Available in *n *= 50.

**Figure 2:**
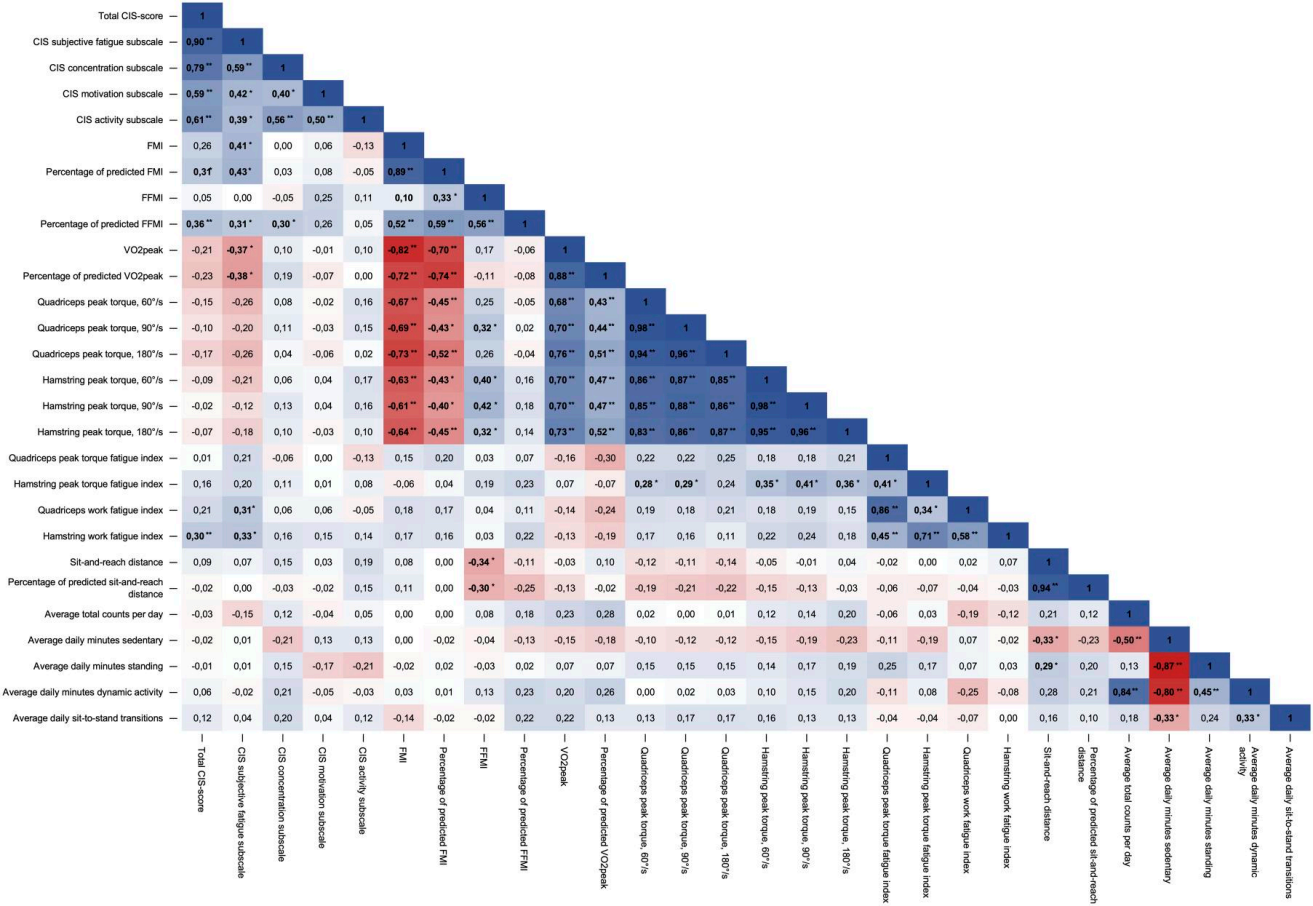
Correlation matrix (Pearson’s *r* or Spearman’s rho) between fatigue, health-related physical fitness, and physical activity. CIS, checklist individual strength; FFMI, fat-free mass index; FMI, fat mass index; VO2peak, oxygen uptake at peak exercise. Significant values are highlighted in bold.* *P* < 0.05, ** *P* < 0.00.

Figure 2 shows the correlation coefficients (Pearson’s *r* or Spearman’s *rho*), organized within a correlation matrix, between measures of fatigue, physical fitness, and physical activity. Significant correlations were observed between the total CIS-score and both the predicted percentage of FMI (*r *= 0.31, *P *< .05) and FFMI (*r *= 0.36, *P *< .001), as well as with the hamstring work fatigue index (*rho *= 0.30, *P *< .001). The CIS subjective fatigue subscale score correlated with FMI and the percentage of predicted FMI (*r *= 0.41 and *r *= 0.43, respectively; *P *< 0.05), FFMI (*r *= 0.31, *P *< .05), VO_2peak_ (*r *=* −*0.37, *P *< .05) and percentage of predicted percentage VO_2peak_ (*r *=* −*0.38, *P *< .05), as well as with quadriceps and hamstring work fatigue indices (*rho *= 0.31 and *rho *= 0.33, respectively; *P *< .05). The CIS concentration subscale score demonstrated a significant correlation with the percentage of predicted FFMI (*rho *= 0.30, *P *< .05). However, the CIS motivation and activity subscale scores did not show significant correlations with any measures of physical fitness or physical activity. No significant correlations were observed between any measures of fatigue and physical activity. Moreover, correlations between physical fitness and physical activity measures were generally weak. The only significant correlation was found between the distance reached with the sit-and-reach test and both the average daily minutes spent sedentary (*r *=* −*0.33, *P *< .05) and standing (*r *= 0.29, *P *< .05).

## Discussion

This study shows that fatigued patients with IBD exhibited higher levels of fat mass, lower cardiorespiratory fitness, and less quadriceps muscular endurance as compared to non-fatigued patients with IBD, indicating that physical deconditioning may play a role in fatigue experienced by patients with IBD. Additionally, various significant correlations were found between fatigue scores and measures of health-related physical fitness. However, even though fatigued patients tended to have lower physical activity levels, no statistically significant differences were observed in physical activity levels between fatigued and non-fatigued patients, and no significant correlations were identified between fatigue scores and physical activity measures.

In this study, approximately one-third of the patients with IBD experienced fatigue complaints, according to the CIS subjective fatigue subscale score. The prevalence of fatigue in previous studies ranged from 53% to 76% for patients with active disease and from 15% to 54% for those in remission.^7^ Considering that the majority of our study population was in clinical and biochemical remission (75% and 85%, respectively), the observed prevalence of fatigue in our cohort aligns with the previously reported prevalence. This underscores that our study population forms a relevant representation of the general IBD population. Additionally, in our study, clinical disease activity was more common among fatigued patients, whereas biochemical disease activity was similar between the 2 groups. These findings are consistent with previous observations that clinical markers of disease activity could be influenced by fatigue complaints (eg, aspects such as general wellbeing), as well as psychological comorbidities prevalent among fatigued patients, thereby affecting the perception and reporting of symptoms and not always accurately reflecting mucosal inflammation.^45^^,^^46^ Moreover, clinical symptoms such as abdominal pain is commonly observed in patients with IBD in remission, often referred to as IBS-like symptoms, and have previously been shown to be associated with psychosocial factors.^47^ However, information on psychological comorbidities was not available in our sample.

The prevalence of overweight and obesity in our cohort was comparable to those reported in previous IBD populations.^48^ Our study population exhibited a predicted FMI percentage of 138.5%, indicating an elevated level of body fat well above the norm. Moreover, the predicted FMI percentage was significantly higher in fatigued patients as compared to non-fatigued patients. Prior research has established an association between obesity and symptoms of fatigue in the general population.^49^^,^^50^ Adipose tissue plays a critical role in the regulation of inflammation by secreting pro-inflammatory cytokines, such as tumor necrosis factor alpha, interleukin-1, and interleuking-6, contributing to a state of low-grade inflammation.^51^ In turn, a low-grade inflammatory state has been proposed to play a role in the pathophysiology of fatigue. Nevertheless, the precise mechanism linking increased adiposity to fatigue remains to be elucidated. We also identified a positive correlation between FFM and measures of fatigue, which could be attributed to the concurrent positive correlation between FMI and FFMI observed in our study. This positive relationship has been described previously and could be explained by an increase in muscle mass to accommodate the additional effort required to support and move the increased adipose tissue.^52^

The median VO_2peak_ of our study population was lower than established normative values for age and sex, achieving on group level only 84.5% of the predicted values. This reduction was significantly more pronounced in patients with fatigue than those without (71.1% vs. 89.6%). Both correlation analysis and multiple linear regression supported these findings, highlighting a robust and independent association between reduced VO_2peak_ and the presence of fatigue. Our findings are consistent with those from a cross-sectional study by Vogelaar *et al.,* which also reported a diminished cardiorespiratory fitness in IBD patients suffering from fatigue.^8^ The authors suggested that this reduced cardiorespiratory fitness could be attributed to the effect of pro-inflammatory cytokines on the brain, resulting in sickness behavior and subsequent decreased physical activity levels. However, despite confirming generally lower activity levels in fatigued patients, our study found no statistically significant differences in measures of physical activity between the fatigued and non-fatigued patients. Additionally, correlation analyses between cardiorespiratory fitness and physical activity levels were low and not significant. However, we did not distinguish between different intensity levels, such as moderate to vigorous physical activity, which has often been linked with enhancements in cardiorespiratory fitness.^53^

Our findings indicate that other factors than the amount of physical activity contribute to the limited VO_2peak_ observed in fatigued patients. One potential contributing factor is that fatigued patients were more frequently active smokers compared to non-fatigued patients. Smoking is known to reduce cardiorespiratory fitness by causing inhaled carbon monoxide to bind to hemoglobin in erythrocytes. This binding effectively displaces oxygen, reducing its transportation to the lungs, muscles, and other vital tissues. Furthermore, the higher smoking prevalence among fatigued patients, together with their lower educational levels, may point to a lower socioeconomic status within this group. Lower socioeconomic status has been linked to poorer cardiorespiratory fitness, often due to less healthy lifestyle behaviors and a higher prevalence of risk factors such as obesity and cardiovascular diseases.^54^ Furthermore, previous research has also demonstrated that lower socioeconomic status is linked to a higher reporting of fatigue symptoms in the general population,^55^^,^^56^ highlighting the importance of considering socioeconomic status as a potential confounding factor in future research.

In our study, we were unable to demonstrate a difference in muscle strength between fatigued and non-fatigued patients, nor did we find significant associations between muscular strength parameters and fatigue. This contrasts with the findings of Vogelaar *et al.,* who found a medium to large effect size for lower muscle strength in fatigued patients as compared to non-fatigued patients with IBD.^8^ A potential explanation for this discrepancy could be that Vogelaar *et al.* did not correct muscular strength for body mass, as we did in our study to obtain a more accurate analysis of muscular strength relative to an individual’s body size. Although it is plausible that muscle strength could be compromised in (a subgroup of) patients with IBD due to the direct effects of pro-inflammatory cytokines or the use of corticosteroids, previous studies on muscle strength in IBD patients have shown controversial findings, warranting further investigation.^19^

Our observations revealed that patients with fatigue had significantly higher quadriceps peak torque fatigue index and a higher work fatigue index, indicative of reduced muscular endurance. Our findings suggest that, although overall muscular strength may not vary significantly between fatigued and non-fatigued patients, the ability of muscles to sustain peak torque or work is notably diminished in those experiencing fatigue. Correlation analysis also revealed a significant relationship between fatigue and both the hamstring and quadriceps work fatigue indices. These findings align with those of Van Langenberg *et al.,* who reported that the rate of muscle fatigue correlated well with subjective self-reported physical fatigue scores in 27 CD patients.^9^ An important explanation for this might be the interaction between the perceived sense of effort and actual performance, a psychophysiological phenomenon that may be more prominent in patients experiencing fatigue.^57^^,^^58^ Fatigued patients might experience increased perceived effort during endurance tasks by influencing the central processing of effort in the brain, potentially leading to earlier discontinuation of the task or reduced performance.

Flexibility, which refers to a range of motion achievable without injury to the joints,^59^ was found to be negatively associated with time spent sedentary and positively with the time spent standing in our study. However, evidence on the link between physical activity and flexibility, as well as its association with health outcomes, such as injuries, lower back pain or mortality remains limited.^59–61^ Therefore, the advantages of improving flexibility in patients with IBD warrants further research.

The results of our study suggest that a decline in physical fitness may play an important role in fatigue experienced by patients with IBD. Enhancing physical fitness may, therefore, be a strategic approach to alleviating these symptoms. Nevertheless, our findings show weak correlations between the levels of physical activity and both fatigue and physical fitness, suggesting that increasing the quantity of physical activity might not significantly impact fatigue or physical fitness levels. Other factors, such as disease-specific or lifestyle-related determinants, may be more predictive of physical fitness and fatigue in patients with IBD. Further research in larger populations is warranted to elucidate these potential predictors and their impact on physical fitness and fatigue in this patient group. Another potential explanation is that a more targeted approach involving tailored exercise, which is a subcategory of physical activity that is planned, repetitive and aiming to improve specific physical fitness components,^13^ may be more effective in managing fatigue in patients with IBD. Several studies have explored the effect of physical activity or physical exercise training programs on fatigue complaints in patients with IBD.^11^^,^^12^^,^^17^ For instance, van Erp *et al.* showed improvements in fatigue and body composition, but not on the primary outcome of cardiorespiratory fitness (ie, maximal oxygen uptake), after a 12-week aerobic exercise regimen.^11^ Furthermore, Lamers *et al.* and McNelly *et al.* examined the effects of advice on increasing physical activity levels. Both studies observed improvements in fatigue complaints, although physical activity levels remained unchanged during the intervention.^12^^,^^17^ The discrepancy between improvements in fatigue outcomes and objective measures of physical fitness or physical activity observed in these studies could be attributable to the Hawthorne effect.^17^ This psychological phenomenon assumes that individuals may alter their behavior or report better outcomes due to the increased attention and awareness from participating in a study, rather than due to the direct effects of the interventions themselves. To deepen our understanding of the relationship between fatigue, physical fitness and physical activity or exercise in IBD, with the aim to enhance management strategies, future studies should adopt a longitudinal design that combines both objective and subjective outcome measures to minimize the influence of the Hawthorne effect. These studies should include accurate measurements of physical fitness, tailored specifically to the purpose of the exercise interventions, enabling researchers to examine whether improvements in physical fitness correlate with better patient-reported outcomes like fatigue.

An important strength of this study is its comprehensive evaluation of all 5 components of physical fitness, including body composition, cardiorespiratory fitness, muscular strength, muscular endurance, and flexibility. These were assessed using gold standard methodologies, ensuring the reliability and validity of the outcomes. Furthermore, data on physical activity was captured through objective accelerometry measurements recorded over several days to provide a detailed evaluation of daily activity levels. In addition, fatigue was measured using the CIS, a well-validated and widely used instrument in Dutch clinical and research settings, including studies in chronic disease populations. The CIS captures multiple dimensions of fatigue (subjective fatigue, concentration, motivation, and activity), which aligns with the multidimensional nature of fatigue in chronic illness, and it includes predefined cutoff scores to identify clinically relevant (subjective) fatigue. To our knowledge, this study is the first to measure all these components of physical fitness in patients with IBD and to explore their relation to fatigue and physical activity. Our study also has limitations. First, the cross-sectional design of this study limits us in establishing causality. Second, although the CIS is widely validated, it was not developed nor validated specifically for IBD, and it does not fully distinguish between physical and cognitive fatigue components. Third, the exploratory nature of the study and its relatively small sample size may have resulted in insufficient power to detect certain significant differences or correlations. Because of the small sample size, were unable to perform a multivariable logistic regression analysis to explore independent predictors of fatigue. Therefore, we decided to perform multiple linear regression analyses to predict fatigue scores while adjusting for key clinical confounders. Third, the limited sample size precluded subgroup analyses by disease activity status. Although biochemical disease activity was comparable between groups and adjusted for in the regression models, stratified analyses could have provided additional insight into the contribution of disease activity to fatigue. Fourth, the sample size also did not allow stratified analyses by disease entity (CD vs. UC). Future studies with larger cohorts should examine whether these findings are consistent across both IBD subtypes. Fifth, to maximize the availability of data on hemoglobin and fecal calprotectin levels, samples collected within an 8-week period around inclusion were utilized. This timeframe was chosen to include as many relevant data points as possible, but it may not accurately reflect the immediate biochemical status of participants at the time of their assessments. Lastly, data on specific nutritional deficiencies (eg, iron and vitamin levels) was not available.

This study showed that reduced physical fitness was associated with fatigue in patients with IBD, suggesting that enhancing physical fitness could help alleviate these symptoms. Physical activity levels were not associated with either fatigue or physical fitness, indicating that simply increasing the amount of physical activity may have limited impact on reducing fatigue or improving physical fitness in patients with IBD. Furthermore, eligible subjects with IBD and fatigue for interventional trials focusing on physical activity should not be based on physical activity levels alone. A more targeted approach involving tailored exercise, aiming to improve specific physical fitness components, may be warranted in managing fatigue in this population. Future longitudinal research should explore the effects of tailored exercise interventions on fatigue and physical fitness in patients with IBD.

## Clinical messages

- Fatigue is a common and debilitating symptom in patients with IBD, but its management remains challenging due to its complex and multifactorial origin.
- Fatigued patients with IBD patients showed significantly higher fat mass, lower cardiorespiratory fitness, and reduced quadriceps muscular endurance, as compared to non-fatigued patients with IBD.
- No associations were found between physical activity levels and either fatigue or physical fitness.
- Improving specific components of physical fitness through tailored physical exercise interventions, rather than promoting general physical activity, may offer an effective strategy for managing fatigue in this population.

## Supplementary Material

otag052_Supplementary_Data

## Data Availability

The datasets generated and analyzed during the present study are available from the corresponding author upon reasonable request.
