# Supplementary material for: Associations between fatigue, physical fitness, and physical activity in patients with inflammatory bowel disease
Source: Crohns Colitis 360. 2026 Jun 12;8(3):otag052. doi: 10.1093/crocol/otag052 (PMC13384068; doi:10.1093/crocol/otag052)
Supplement: otag052_Supplementary_Data [file otag052_supplementary_data.docx]

**Supplemental file**

**Supplemental Table I.** Unadjusted and adjusted linear regression models predicting the total CIS score

|  | Unadjusted | |  | Adjusted^§^ | | |
| --- | --- | --- | --- | --- | --- | --- |
|  | B | 95% CI | p-value | B | 95% CI | p-value |
| FMI (kg/m^2^)^†^ | 1.68 | -0.12 – 3.49 | *p* = 0.067 | 1.45 | -0.61 – 3.51 | *p* = 0.163 |
| FFMI (kg/m^2^)^†^ | 0.95 | -1.76 – 3.67 | *p* = 0.483 | 1.77 | -1.21 – 4.75 | *p* = 0.237 |
| VO_2peak_ (ml/kg/min)^†^ | -0.50 | -1.17 – 0.17 | *p* = 0.138 | -0.55 | -1.35 -0.26 | *p* = 0.178 |
| Quadriceps peak torque, 60°/s (Nm/kg) | -5.77 | -16.83 – 5.29 | *p* = 0.300 | -4.64 | -20.62 – 11.33 | *p* = 0.561 |
| Quadriceps peak torque, 90°/s (Nm/kg) | -4.79 | -18.10 – 8.53 | *p* = 0.474 | -0.37 | -21.87 – 21.12 | *p* = 0.972 |
| Quadriceps peak torque, 180°/s (Nm/kg) | -11.31 | -29.26 – 6.64 | *p* = 0.212 | -13.94 | -41.85 – 13.98 | *p* = 0.319 |
| Hamstring peak torque, 60°/s (Nm/kg) | -6.39 | -25.87 – 13.09 | *p* = 0.513 | -11.87 | -37.31 – 13.58 | *p* = 0.941 |
| Hamstring peak torque, 90°/s (Nm/kg) | -1.70 | -23.09 – 19.70 | *p* = 0.874 | -5.33 | -35.07 – 24.40 | *p* = 0.719 |
| Hamstring peak torque, 180°/s (Nm/kg) | -7.15 | -34.81 – 20.52 | *p* = 0.606 | -14.13 | -50.97 – 22.70 | *p* = 0.443 |
| Quadriceps peak torque fatigue index | 0.35 | -0.28 – 0.98 | *p* = 0.267 | 0.01 | -0.56 – 0.58 | *p* = 0.974 |
| Hamstring peak torque fatigue index | 0.32 | -0.34 – 0.97 | *p* = 0.334 | 0.09 | -0.48 – 0.67 | *p* = 0.749 |
| Quadriceps work fatigue index | 0.28 | -0.06 – 0.61 | *p* = 0.109 | 0.12 | -0.19 – 0.43 | *p* = 0.429 |
| Hamstring work fatigue index | 0.48 | -0.03 – 0.99 | *p* = 0.064 | 0.32 | -0.13 – 0.77 | *p* = 0.156 |
| Sit-and-reach test (cm) | 0.22 | -0.45 – 0.90 | *p* = 0.511 | -0.03 | -0.64 – 0.59 | *p* = 0.933 |
| *Physical activity*^‡^ |  |  |  |  |  |  |
| Average daily counts per second | 0.00 | 00.00 – 0.00 | *p* = 0.816 | 0.00 | 00.00 – 0.00 | *p* = 0.414 |
| Average daily minutes sedentary | -0.01 | -0.09 – 0.08 | *p* = 0.896 | 0.01 | -0.06 – 0.08 | *p* = 0.744 |
| Average daily minutes standing | -0.01 | -0.13 – 0.11 | *p* = 0.930 | -0.02 | -0.12 – 0.08 | *p* = 0.712 |
| Average daily minutes dynamic activity | 0.03 | -0.13 – 0.19 | *p* = 0.706 | -0.01 | -0.14 – 0.12 | *p* = 0.874 |
| Average daily sit-to-stand transitions | 0.04 | -0.02 – 0.10 | *p* = 0.151 | 0.03 | -0.02 – 0.08 | *p* = 0.171 |

Abbreviations: CI = confidence interval, CIS = checklist individual strength, CPET = cardiopulmonary exercise test, FFMI = fat-free mass index, FMI = fat mass index, VO_2peak_ = oxygen uptake at peak exercise. ^†^ Available in *n* = 52; ^‡^ Available in *n* = 5; ^§^ adjusted for age, sex, active smoking, and faecal calprotectin^.^

**Supplemental Table II.** Unadjusted and adjusted linear regression models predicting the CIS concentration subscale score

|  | Unadjusted | |  | Adjusted^§^ | | |
| --- | --- | --- | --- | --- | --- | --- |
|  | B | 95% CI | p-value | B | 95% CI | p-value |
| FMI (kg/m^2^)^†^ | 0.13 | -0.50 – 0.76 | *p* = 0.689 | -0.09 | -0.88 – 0.70 | *p* = 0.818 |
| FFMI (kg/m^2^)^†^ | -0.06 | -0.98 – 0.87 | *p* = 0.904 | 0.31 | -0.83 – 1.44 | *p* = 0.586 |
| VO_2peak_ (ml/kg/min)^†^ | 0.00 | -0.23 – 0.23 | *p* = 0.974 | 0.10 | -0.21 – 0.41 | *p* = 0.507 |
| Quadriceps peak torque, 60°/s (Nm/kg) | 0.04 | -3.71 – 3.80 | *p* = 0.981 | 1.38 | -4.59 – 7.35 | *p* = 0.644 |
| Quadriceps peak torque, 90°/s (Nm/kg) | 0.56 | -3.92 – 5.07 | *p* = 0.798 | 3.87 | -4.06 – 11.80 | *p* = 0.330 |
| Quadriceps peak torque, 180°/s (Nm/kg) | -0.60 | -6.72 – 5.52 | *p* = 0.845 | 1.34 | -9.19 – 11.87 | *p* = 0.799 |
| Hamstring peak torque, 60°/s (Nm/kg) | 0.29 | -6.28 – 6.85 | *p* = 0.931 | 0.06 | -9.53 – 9.65 | *p* = 0.990 |
| Hamstring peak torque, 90°/s (Nm/kg) | 2.10 | -5.06 – 9.26 | *p* = 0.559 | 3.70 | -7.35 – 14.75 | *p* = 0.503 |
| Hamstring peak torque, 180°/s (Nm/kg) | 1.86 | -7.44 – 11.15 | *p* = 0.690 | 3.08 | -10.72 – 16.89 | *p* = 0.655 |
| Quadriceps peak torque fatigue index | 0.03 | -0.19 – 0.24 | *p* = 0.791 | -0.09 | -0.30 – 0.13 | *p* = 0.426 |
| Hamstring peak torque fatigue index | 0.06 | -0.17 – 0.28 | *p* = 0.613 | -0.01 | -0.22 – 0.21 | *p* = 0.939 |
| Quadriceps work fatigue index | 0.04 | -0.07 – 0.16 | *p* = 0.467 | -0.02 | -0.13 – 0.10 | *p* = 0.799 |
| Hamstring work fatigue index | 0.07 | -0.10 – 0.25 | *p* = 0.400 | 0.02 | -0.15 – 0.19 | *p* = 0.808 |
| Sit-and-reach test (cm) | 0.10 | -0.13 – 0.32 | *p* = 0.392 | -0.03 | -0.26 – 0.20 | *p* = 0.822 |
| *Physical activity^‡^* |  |  |  |  |  |  |
| Average daily counts per second | 0.00 | 0.00 – 0.00 | *p* = 0.084 | 0.00 | 0.00 – 0.00 | *p* = 0.559 |
| Average daily minutes sedentary | 0.00 | -0.05 – 0.00 | *p* = 0.083 | -0.02 | -0.04 – 0.01 | *p* = 0.192 |
| Average daily minutes standing | 0.03 | -0.02 – 0.07 | *p* = 0.208 | 0.02 | -0.02 – 0.06 | *p* = 0.342 |
| Average daily minutes dynamic activity | 0.05 | -0.01 – 0.10 | *p* = 0.078 | 0.03 | -0.02 – 0.08 | *p* = 0.189 |
| Average daily sit-to-stand transitions | 0.02 | 0.00 – 0.04 | ***p* = 0.035** | 0.02 | 0.00 – 0.04 | *p* = 0.062 |

Abbreviations: CI = confidence interval, CIS = checklist individual strength, CPET = cardiopulmonary exercise test, FFMI = fat-free mass index, FMI = fat mass index, VO_2peak_ = oxygen uptake at peak exercise. ^†^ Available in *n* = 52; ^‡^ Available in *n* = 5; ^§^ adjusted for age, sex, active smoking, and faecal calprotectin.

**Supplemental Table III.** Unadjusted and adjusted linear regression models predicting the CIS motivation subscale score

|  | Unadjusted | |  | Adjusted^§^ | | |
| --- | --- | --- | --- | --- | --- | --- |
|  | B | 95% CI | p-value | B | 95% CI | p-value |
| FMI (kg/m^2^)^†^ | 0.22 | -0.15 – 0.58 | *p* = 0.238 | 0.42 | -0.08 – 0.92 | *p* = 0.095 |
| FFMI (kg/m^2^)^†^ | 0.36 | -0.16 – 0.886 | *p* = 0.173 | 0.70 | -0.01 – 1.40 | *p* = 0.053 |
| VO_2peak_ (ml/kg/min)^†^ | -0.07 | -0.20 – 0.07 | *p* = 0.315 | -0.16 | -0.35 – 0.04 | *p* = 0.115 |
| Quadriceps peak torque, 60°/s (Nm/kg) | -0.80 | -2.96 – 1.35 | *p* = 0.459 | -1.77 | -5.60 – 2.06 | *p* = 0.357 |
| Quadriceps peak torque, 90°/s (Nm/kg) | -1.03 | -3.61 – 1.55 | *p* = 0.426 | -3.01 | -8.11 – 2.10 | *p* = 0.241 |
| Quadriceps peak torque, 180°/s (Nm/kg) | -1.68 | -5.18 – 1.83 | *p* = 0.341 | -4.40 | -11.08 – 2.28 | *p* = 0.191 |
| Hamstring peak torque, 60°/s (Nm/kg) | -0.56 | -4.35 – 3.23 | *p* = 0.768 | -1.27 | -7.46 – 4.92 | *p* = 0.681 |
| Hamstring peak torque, 90°/s (Nm/kg) | -0.77 | -4.91 – 3.37 | *p* = 0.711 | -2.46 | -9.61 – 4.68 | *p* = 0.491 |
| Hamstring peak torque, 180°/s (Nm/kg) | -1.83 | -7.18 – 3.52 | *p* = 0.495 | -4.14 | -13.00 – 4.72 | *p* = 0.351 |
| Quadriceps peak torque fatigue index | -0.03 | -0.16 – 0.09 | *p* = 0.586 | -0.04 | -0.18 – 0.09 | *p* = 0.523 |
| Hamstring peak torque fatigue index | -0.05 | -0.17 – 0.08 | *p* = 0.476 | -0.04 | -0.18 – 0.10 | *p* = 0.535 |
| Quadriceps work fatigue index | -0.02 | -0.09 – 0.05 | *p* = 0.586 | -0.03 | -0.10 – 0.05 | *p* = 0.496 |
| Hamstring work fatigue index | 0.02 | -0.09 – 0.12 | *p* = 0.765 | 0.02 | -0.09 – 0.13 | *p* = 0.708 |
| Sit-and-reach test (cm) | -0.02 | -0.16 – 0.11 | *p* = 0.718 | -0.06 | -0.20 – 0.09 | *p* = 0.442 |
| *Physical activity^‡^* |  |  |  |  |  |  |
| Average daily counts per second | 0.00 | 0.00 – 0.00 | *p* = 0.882 | 0.00 | 0.00 – 0.00 | *p* = 0.741 |
| Average daily minutes sedentary | 0.01 | -0.01 – 0.02 | *p* = 0.320 | 0.01 | -0.01 – 0.03 | *p* = 0.182 |
| Average daily minutes standing | -0.02 | -0.04 – 0.01 | *p* = 0.200 | -0.02 | -0.04 – 0.01 | *p* = 0.119 |
| Average daily minutes dynamic activity | 0.00 | -0.04 – 0.03 | *p* = 0.782 | -0.01 | -0.04 – 0.02 | *p* = 0.561 |
| Average daily sit-to-stand transitions | 0.00 | -0.01 – 0.02 | *p* = 0.440 | 0.01 | -0.01 – 0.02 | *p* = 0.461 |

Abbreviations: CI = confidence interval, CIS = checklist individual strength, CPET = cardiopulmonary exercise test, FFMI = fat-free mass index, FMI = fat mass index, VO_2peak_ = oxygen uptake at peak exercise. ^†^ Available in *n* = 52; ^‡^ Available in *n* = 50; ^§^ adjusted for age, sex, active smoking, and faecal calprotectin.

**Supplemental Table IV.** Unadjusted and adjusted linear regression models predicting the CIS activity subscale score

|  | Unadjusted | |  | Adjusted^§^ | | |
| --- | --- | --- | --- | --- | --- | --- |
|  | B | 95% CI | p-value | B | 95% CI | p-value |
| FMI (kg/m^2^)^†^ | -0.12 | -0.42 – 0.19 | *p* = 0.447 | -0.06 | -0.49 – 0.37 | *p* = 0.790 |
| FFMI (kg/m^2^)^†^ | 0.08 | -0.36 – 0.53 | *p* = 0.708 | -0.04 | -0.66 – 0.57 | *p* = 0.892 |
| VO_2peak_ (ml/kg/min)^†^ | 0.04 | -0.07 – 0.15 | *p* = 0.488 | -0.01 | -0.18 – 0.15 | *p* = 0.872 |
| Quadriceps peak torque, 60°/s (Nm/kg) | 0.70 | -1.10 – 2.50 | *p* = 0.438 | 0.48 | -2.76 – 3.72 | *p* = 0.766 |
| Quadriceps peak torque, 90°/s (Nm/kg) | 0.86 | -1.30 – 3.01 | *p* = 0.429 | 0.41 | -3.94 – 4.75 | *p* = 0.851 |
| Quadriceps peak torque, 180°/s (Nm/kg) | 0.34 | -2.62 – 3.29 | *p* = 0.820 | -1.97 | -7.64 – 3.71 | *p* = 0.489 |
| Hamstring peak torque, 60°/s (Nm/kg) | 1.77 | -1.37 – 4.90 | *p* = 0.263 | 1.20 | -3.99 – 6.38 | *p* = 0.644 |
| Hamstring peak torque, 90°/s (Nm/kg) | 1.80 | -1.63 – 5.23 | *p* = 0.297 | 0.69 | -5.33 – 6.71 | *p* = 0.818 |
| Hamstring peak torque, 180°/s (Nm/kg) | 2.35 | -2.10 – 6.80 | *p* = 0.294 | 1.44 | -6.04 – 8.93 | *p* = 0.700 |
| Quadriceps peak torque fatigue index | -0.02 | -0.13 – 0.08 | *p* = 0.657 | -0.05 | -0.16 – 0.07 | *p* = 0.390 |
| Hamstring peak torque fatigue index | 0.03 | -0.08 – 0.13 | *p* = 0.621 | 0.01 | -0.1 – 0.13 | *p* = 0.819 |
| Quadriceps work fatigue index | 0.02 | -0.04 – 0.07 | *p* = 0.574 | 0.01 | -0.05 – 0.07 | *p* = 0.756 |
| Hamstring work fatigue index | 0.06 | -0.02 – 0.15 | *p* = 0.145 | 0.07 | -0.02 – 0.16 | *p* = 0.116 |
| Sit-and-reach test (cm) | 0.06 | -0.05 – 0.17 | *p* = 0.309 | 0.05 | -0.07 – 0.18 | *p* = 0.379 |
| *Physical activity^‡^* |  |  |  |  |  |  |
| Average daily counts per second | 0.00 | 0.00 – 0.00 | *p* = 0.792 | 0.00 | 0.00 – 0.00 | *p* = 0.945 |
| Average daily minutes sedentary | 0.01 | 0.00 – 0.02 | *p* = 0.156 | 0.01 | 0.00 – 0.03 | *p* = 0.062 |
| Average daily minutes standing | -0.02 | -0.04 – 0.00 | *p* = 0.085 | -0.02 | -0.04 – 0.00 | ***p* = 0.037** |
| Average daily minutes dynamic activity | -0.01 | -0.03 – 0.02 | *p* = 0.591 | -0.01 | -0.04 – 0.01 | *p* = 0.345 |
| Average daily sit-to-stand transitions | 0.00 | -0.01 – 0.01 | *p* = 0.453 | 0.00 | -0.01 – 0.01 | *p* = 0.905 |

Abbreviations: CI = confidence interval, CIS = checklist individual strength, CPET = cardiopulmonary exercise test, FFMI = fat-free mass index, FMI = fat mass index, VO_2peak_ = oxygen uptake at peak exercise. ^†^ Available in *n* = 52; ^‡^ Available in *n* = 50; ^§^ adjusted for age, sex, active smoking, and faecal calprotectin.
